# Functional Hypergraphs of Stock Markets

**DOI:** 10.3390/e26100848

**Published:** 2024-10-08

**Authors:** Jerry Jones David, Narayan G. Sabhahit, Sebastiano Stramaglia, T. Di Matteo, Stefano Boccaletti, Sarika Jalan

**Affiliations:** 1 Complex Systems Lab, Department of Physics, Indian Institute of Technology Indore, Khandwa Road, Indore 453552, India; jerrydavid8@gmail.com; 2 Network Science Institute, Northeastern University, Boston, MA 02115, USA; narayan.g.sabhahit@gmail.com; 3 Dipartimento Interateneo di Fisica, Universitá degli Studi di Bari Aldo Moro and INFN, 70125 Bari, Italy; sebastiano.stramaglia@ba.infn.it; 4 Department of Mathematics, King’s College London, Strand, London WC2R 2LS, UK; dimatteo.tiz@gmail.com; 5 Museo Storico della Fisica e Centro Studi e Ricerche Enrico Fermi, Via Panisperna 89 A, 00184 Rome, Italy; 6 Complexity Science Hub Vienna, Josefstädter Straße 39, 1080 Vienna, Austria; 7 CNR—Institute of Complex Systems, Via Madonna del Piano 10, 50019 Sesto Fiorentino, Italy; stefano.boccaletti@gmail.com

**Keywords:** hypergraphs, stock markets, complex systems

## Abstract

In stock markets, nonlinear interdependencies between various companies result in nontrivial time-varying patterns in stock prices. A network representation of these interdependencies has been successful in identifying and understanding hidden signals before major events like stock market crashes. However, these studies have revolved around the assumption that correlations are mediated in a pairwise manner, whereas, in a system as intricate as this, the interactions need not be limited to pairwise only. Here, we introduce a general methodology using information-theoretic tools to construct a higher-order representation of the stock market data, which we call *functional hypergraphs*. This framework enables us to examine stock market events by analyzing the following functional hypergraph quantities: Forman–Ricci curvature, von Neumann entropy, and eigenvector centrality. We compare the corresponding quantities of networks and hypergraphs to analyze the evolution of both structures and observe features like robustness towards events like crashes during the course of a time period.

## 1. Introduction

The past two decades have seen a spurt in applications of complex network methodologies in deciphering properties of underlying complex systems [1,2]. A network consists of nodes and edges, with the nodes representing subsystems or units of the system and the edges representing interactions between them. A considerable number of studies on network topologies of various real-world complex systems have been conducted [3,4,5] since the landmark paper of Barabási and Albert revealing that many real-world networks are scale-free [6], i.e., the degree distribution of a diverse range of real networks follows power-law rather than, for example, the Poisson distribution, as in Erdös-Rényi random networks [7]. Also, a number of studies have been directed at the geometry of networks and filtering of edges according to their importance in a dense network [8,9,10]. Various structural and spectral properties of complex networks are known to be closely linked with dynamical processes such as synchronization [11]. So far, investigations of complex systems have primarily revolved around the assumption that their units communicate through pairwise interactions; however, recently, it has been shown that various structural and dynamical properties of many real-world complex systems are often defined and governed by higher-order or symplectic interactions [12,13,14,15]. Network representation, by definition, is only able to model pairwise interactions. To incorporate simplicial complexes and higher-order interactions, hypergraphs are being introduced [16]. Here, we set out to study the existence of higher-order interactions in real-world complex systems by considering the examples of stock markets. Many previous works have studied the underlying pairwise network structures of various stock markets [5,17,18,19,20]. By considering Pearson correlation or mutual information, these works have focused on constructing pairwise networks to represent the underlying interactions. However, despite the success of the traditional dyadic representation of coupling, it fails to capture interaction patterns among the nodes in many real-world systems. Recently, many diverse systems such as collaboration networks [21], brain networks (structural [22], functional [12]), cellular networks [23], and ecosystems [24] have revealed the existence of interactions occurring among more than two nodes at a time. These interactions involving three or more nodes, known as higher-order interactions, are represented using a hypergraph or simplicial complexes [16].

So far, studies on higher-order interactions have embarked on their way into several real-world networks. For example, Hergoz et al. [25] developed a higher-order functional connectivity method to capture interactions among three or more brain regions across spatiotemporal scales and studied detailed EEG and fMRI-compatible characterizations of neurodegenerative conditions. Next, the neuronal studies of Chelaru et al. [26] showed that pairwise interactions between neurons explain the population response in sensory areas. In contrast, higher-order interactions explain the responses in executive areas encoding decision making and actions.

Santoro et al. [27] analyzed multivariate time series and studied the higher-order patterns in real-world data of brain functional activity, epidemics, and financial markets. Further, in ref. [27], the authors developed a multivariate signal processing method to construct higher-order networks from fMRI time series. They applied it to resting fMRI signals to identify higher-order communications among brain regions. Faes et al. [28] defined a new metric O−information rate to evaluate higher-order interactions across multivariate time series. They applied this approach to physiological networks described by heart period, respiration variability, and arterial pressure of healthy subjects and to brain networks described by an electrocorticographic signal obtained in an animal experiment during anesthesia. Cencetti et al. analyzed higher-order organizations, their formations, and evolution in temporal networks by studying five social network data sets from different social settings. Also, in ref. [29], the authors proposed STHAN-SR, a neural hypergraph architecture for stock selection. They modeled the complex relations among stocks through a hypergraph and temporal Hawkes attention mechanism. By doing so, the authors tailored a new spatiotemporal attention hypergraph network architecture to rank stocks based on profit by jointly modeling stock interdependence and the temporal evolution of their prices.

Here, we present results for synergistic higher-order interactions for five stock markets: BSE from India, DAX from Germany, FTSE from Britain, NIKKEI from Japan, and SP500 from the USA. By considering the time series of the stocks, we analyze how structural properties of hypergraphs of higher-order interactions capture events of the underlying systems. Also, in addition to the similarity measures we have chosen, the extension to higher-order interactions also naturally encompasses nonlinear types of dependencies by going beyond pairwise interactions.

This paper is broadly divided into the following sections. The Section 2 provides the basics of information theory and graph construction framework using mutual and interaction information. The Section 3 gives a complete description of the data and their preprocessing to obtain the actual data we work with. Also, we discuss the methodology by which networks and hypergraphs are obtained. The Section 4 calculates and analyses network quantities, which could give insight into and act as indicators of actual events in the market.

## 2. Methodology

### 2.1. Information-Theoretic Basics

Stemming from Shannon’s groundbreaking work [30], information theory has come a long way in helping to understand interactions in complex systems. Shannon entropy quantifies the amount of inherent uncertainty in the outcome of a random variable,

$$H\left(X\right)=-\sum_{x\in X}P\left(x\right)\log\left(P\left(x\right)\right).$$

Mutual information (MI) I(X;Y) provides a measure of the information shared between two variables (say *X* and *Y*), or in other words, MI quantifies the expected information that is obtained about the state of *Y* by observing the state of *X*. MI takes the form of Kullback–Leibler divergence between the joint probability and the product of its marginals. MI is symmetric and non-negative and is 0 for two independent probability distributions. MI quantifies information shared between two variables or two sets of variables.

(1) $$I\left(X;Y\right)=\sum_{x\in X}\sum_{y\in Y}P\left(x,y\right)\log\left(\frac{P(x,y)}{P(x)P(y)}\right).$$

As seen from Equation (1), mutual information is a function of the marginal and joint probability distributions of the random variables *X* and *Y*. There have been multiple attempts to generalize mutual information for more than two variables. A few well-known methods are based on total correlation [31] and interaction information [32]. The total correlation measure simply represents the overall dependency between the variables and reveals nothing about how these dependencies are distributed among the subset of variables. On the other hand, interaction information was proposed as a measure to decipher the amount of information shared among a set of variables that are not shared among any of its subsets. Hence, here, we focus on interaction information, which, for three variables (X,Y,Z), takes the following form:

(2) I(X;Y;Z)=I(X;Y)−I(X;Y|Z).

where I(X;Y|Z) is called conditional mutual information (CMI) [33]. CMI quantifies how a third variable *Z* affects the information shared between *X* and *Y*,

$$\begin{array}{cc} I(X;Y|Z) & =\sum_{z\in Z}P\left(z\right)\sum_{x\in X}\sum_{y\in Y}P\left(x,y|z\right)\log\left(\frac{P(x,y|z)}{P(x|z)P(y|z)}\right). \end{array}$$

CMI can be further broken down and written in terms of mutual information terms,

$$\begin{array}{cc} I(X;Y|Z) & =\sum_{z\in Z}\sum_{x\in X}\sum_{y\in Y}P\left(x,y,z\right)\log\left(\frac{P(x,y,z)P(z)}{P(x,z)P(y,z)}\right)\\ \; & =\sum_{z\in Z}\sum_{x\in X}\sum_{y\in Y}P\left(x,y,z\right)\left(\log(P(x,y,z)+\log(P(z))-\log(P(x,z))-\log(P(y,z))\right)\\ \; & =H(X,Y,Z)+H(Z)-H(X,Z)-H(Y,Z)+H(X)-H(X)\\ \; & =I(X;Y,Z)-I(X;Z). \end{array}$$

I(X;Y,Z) is referred to as the total information, and the relation I(X;Y,Z)=I(X;Z)+I(X;Y|Z) is called the chain rule of the mutual information. Plugging this simplification into Equation (2), we arrive at the following equation:

(3) I(X;Y;Z)=I(X;Y)+I(X;Z)−I(X;Y,Z).

The interaction information is a symmetric measure, i.e., any permutation of the input variables yields the same output as each term on the right-hand side represents mutual information between two quantities. Therefore, in Equation (2), it does not matter what variable we condition on. However, interaction information can take both negative and positive values, unlike mutual information. A deep insight into the inference of interaction information values is provided in ref. [34]. It was proposed that the total information for the case of three variables can be broken down into four contributions: the unique information that *Z* provides about *X*, U(X;Z∖Y), the unique information that *Y* provides about *X*, U(X;Y∖Z), and the common information that both *Y* and *Z* provide about *X*, called redundant information (R(X;Y,Z)), which is nothing but U1(X:Y∖Z)∩U2(X:Z∖Y). Redundant information corresponds to the information obtained about *X* by observing the variables (Y,Z) together, the same as the information obtained about *X* by independently observing each source variables *Y* and *Z*. Finally, some information about *X* is obtained only when we consider *Y* and *Z* jointly. This part of the information is termed synergistic information and denoted as S(X;Y,Z).

**Figure 1:**
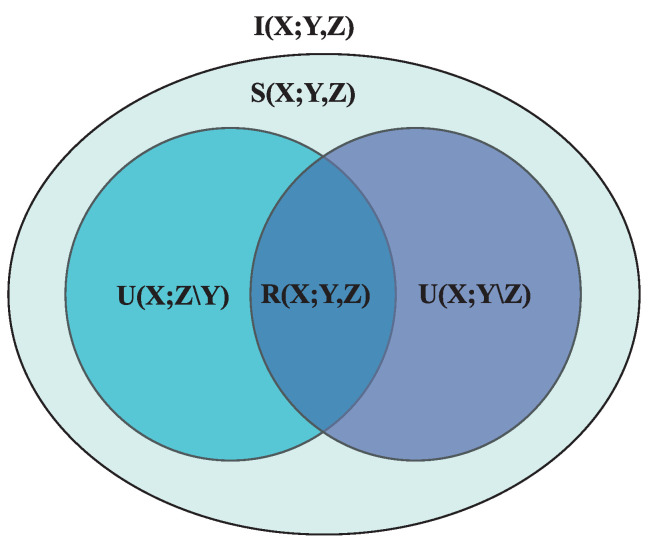
Schematic for multivariate information for three variables as proposed in [34]. The total information I(X;Y,Z) is the sum of the unique information provided by *Z* about *X*, U(X;Z∖Y), the unique information provided by *Y* about *X*, U(X;Y∖Z), the redundant information R(X;Y,Z), and the synergistic information S(X;Y,Z). The redundant information refers to the same information about *X* that both *Y* and *Z* give. Synergistic information refers to the information about *X* obtained only when we take *Y* and *Z* together.

As illustrated by Figure 1, the total information provided by the source variables *Y* and *Z* about *X*, the mutual information between *X* and *Y*, and the mutual information between *X* and *Z* take the following form:

$$\begin{array}{c} I(X;Y,Z)=U(X;Z\setminus Y)+U(X;Y\setminus Z)+R(X;Y,Z)+S(X;Y,Z)\\ I(X;Z)=U(X;Z\setminus Y)+R(X;Y,Z)\\ I(X;Y)=U(X;Y\setminus Z)+R(X;Y,Z) \end{array}$$

By using these expressions of the mutual information, the interaction information of Equation (3) can be written as

I(X;Y;Z)=R(X;Y,Z)−S(X;Y,Z).

The interaction information corresponds to the difference between the redundant and synergistic information and can be a drawback for systems with synergistic and redundant interactions. Therefore, the result from interaction information might be confounding. The only way to explicitly decouple and obtain a system’s values of synergy and redundancy is to define any unique, redundant, or synergistic information terms. We point out that this has been a very active area of research in the community, and we refer the reader to a recent survey on the same [35]. Nevertheless, the value of interaction information provides an idea of which kind of interaction dominates. A negative value of interaction information means that the three variables under consideration are predominantly synergistic, whereas a positive value indicates redundancy. A detailed study of the interaction information to detect synergistic interactions in synthetically constructed data is carried out in ref. [36]. Although it considers a newly defined measure called the O-information [37], we note that O-information reduces to interaction information for the case of three variables.

Apart from the advantage that the extension of the same quantity can be used for both pairwise and three-body interactions, mutual information and interaction information have one more common advantage over the conventionally used Pearson correlation because of their ability to detect nonlinear correlations too [38]. This paper only focuses on triadic higher-order interactions; however, the proposed methodology can be straightforward extendable to other higher orders (quadratic, etc.) of interactions.

Next, armed with the tools borrowed from the information theory, we set out to understand pairwise and higher-order interactions in the stock markets. Let us first make the terms “pairwise interactions” and “higher-order interactions” precise for the system considered here before we start using them. The interactions between the subsystems can mean different things for different complex systems. In the context of the stock market, we identify pairwise interaction between, say, variables *X* and *Y*, as the expected information that is obtained about the state of *Y* by observing the state of *X*, which is nothing but the mutual information between *X* and *Y*. Also, for variables XY,Z, the redundant information dominates the synergistic information if the interaction information is positive. This means there is no new information about the target variable *X* when we observe the source variables together, Y,Z, compared with when we observe the source variables independently. Hence, we conclude that pairwise interactions can represent such a collection of variables. On the contrary, suppose interaction information turns out to be negative, indicating predominantly synergistic information, which means that observing any of the two of the three source variables together gives us new information about the third. We coin cases where synergistic information dominates as synergistic or higher-order interactions of order three. Also, to ensure the statistical significance of these results, we perform p test for each value of mutual information and interaction information as shown in Figure A1.

Next, to compare the time variability of the network structure and hypergraph, we need to ensure that the same threshold is considered across all the windows for constructing both the pairwise networks and the hypergraphs. Here, we consider the threshold as the mean of the distribution of nonzero edge weights and calculate it for mutual information for each window to construct the network. The triadic interactions do not persist long in the sense that the structure of the hypergraph changes in a short period. In contrast, the network structure persists for a much longer time scale.

Also, to ensure the robustness of the findings to variations in the choice of thresholds and time windows, we look into both the overall network behavior in Figure A2 and individual node behavior to the variation in threshold value in Figure A3. As for the time windows, we construct the networks and hypergraphs with different choices of time windows and plot the time evolution of the number of edges in Figure A4. These three figures ensure that our results are robust to the variation in window size and threshold.

To examine the network and hypergraph structures more deeply and to analyze whether they yield something significant that is not immediately obvious from the primary network quantities, like the degree of nodes and the average value of mutual information and interaction information, we also calculate two more quantities, Forman–Ricci curvature and von Neumann entropy, which in the case of the pairwise network has shown to be correlated with actual phenomena that happen in the market.

### 2.2. Forman–Ricci Curvature

The curvature of a manifold is a well-established quantity in physics. It has been used extensively in general relativity. The value of Ricci curvature at a point quantifies how the local geometry of that point varies from that of an Euclidian manifold [39]. The concept of Ricci curvature has been extended to networks, and more than one analog of the same exists, namely Ollivier–Ricci curvature, Menger–Ricci curvature, Haantjer–Ricci curvature, and Forman–Ricci curvature [20]. As each is an extension of a quantity defined on a manifold, each takes different assumptions and is suited for analyzing various network properties. In this work, we use Forman–Ricci curvature, defined by Robin Forman in [40], which was brought into complex networks by [41]. We use Forman–Ricci curvature of an edge for an unweighted network, as proposed in ref. [42] as follows:

(4) $${F_{e}}^{2}=4-d_{2}^{i}-d_{2}^{j},$$

where *i* and *j* are the node indices of those nodes connected by the edge *e* and $d_{2}^{i}$ is the degree of the *i*th node. Equation (4) comes from a more general form of the expression for a weighted network in [42]. Regarding implications for networks, we can think it is a measure of how a network grows from an edge, just like how a surface expands or contracts from a point given by the Ricci curvature for surfaces. This quantity has been presented as an indicator of market instability in ref. [20]. Here, we use an analog of Forman–Ricci curvature, defined in [42],

(5) $${F_{e}}^{3}=6-d_{3}^{i}-d_{3}^{j}-d_{3}^{k},$$

where *i*, *j*, and *k* are the node indices of those nodes connected by the hyperedge *e* and $d_{3}^{i}$ is the hyperdegree of the *i*th node. Equation (5) gives the higher-order curvature as a function of edge degrees. Similar to the pairwise curvature quantifying the density of a network into a hyperbolic structure, it is reasonable to assume that its higher-order analogue tells a similar story. There are a few windows in which hypergraphs are absent. We consider the curvature in those windows to be zero. The formula shows that as the number of connections of a node increases, FRC will have a more negative value. Therefore, community formation will decrease FRC’s negative value corresponding to intra- and intercommunity edges. However, for an intracommunity link, even when there is a high negative value of FRC, the connections will be localized in a set of nodes; so, in a sense, FRC does not give information about the growth at those edges, whereas, in the case of an intercommunity edge, it does. Moreover, FRC, from an intracommunity edge, is a more appropriate measure for the co-evolution of those companies in the community.

### 2.3. Von Neumann Entropy

Von Neumann entropy extends classical Gibbs entropy to a quantum statistical system [43]. It uses the density matrix formulation of a quantum system, which especially suits a quantum many-body system. It works for a mixed system, a system that exists as a statistical mixture of different quantum states and measures how two mixed systems differ. Extending this many-body approach to networks, ref. [44] interpreted the Laplacian as the density matrix of a graph and used the analogy to define von Neumann entropy for networks. Analogous to von Neumann entropy quantifying the mixedness of a many-body quantum system, the von Neumann entropy for networks quantifies whether a network is pure. A pure network is one whose Laplacian is of the particular form [44]. Also, von Neumann’s entropy for graphs has a profound connection with the number of connected components via its eigenvalues. Von Neumann entropy has connections with the network structure. Hence, in our case, the evolution of the market and its network structure accounts for the variation in von Neumann entropy. We follow [44] to calculate the von Neumann entropy for networks as

(6) $$S=-\sum_{i=1}^{n}\lambda_{i}\left(\rho \right)\log\lambda_{i}\left(\rho \right).$$

Equation (6) gives the von Neumann entropy of the network in terms of eigenvalues of the adjacency matrix. However, the extension for the same for hypergraphs was taken from [45], which was performed using higher-order singular value decomposition (HOSVD), where the singular values of the adjacency tensor are calculated after unfolding it into a rectangular matrix.

## 3. Data

We applied the dynamic analysis to the time series data of five markets consisting of stocks’ adjusted daily closing price from 8 February 2010 to 25 March 2022. The adjusted closing price data of stocks are obtained from Bloomberg. We have a time series of length 3145 for each stock in each market. We chose 26 of the most capitalized stocks from each market. For analysis, 30 sliding windows are considered in the time series of adjusted close price, each with a length of 200 days and an overlap of 100 days between the consecutive windows. We denote window index with *i*.

Let $X_{l}=\left(x_{1},x_{2},\ldots ,x_{t},\ldots ,x_{3145}\right)$ be the adjusted close time series corresponding to the stock *l*; then, from the adjusted price time series, we calculate the log returns time series,

(7) $$r_{t}=\log\left(x_{t+1}\right)-\log\left(x_{t}\right)\;|\;t\in \left[1,3145\right].$$

We start by estimating mutual information between the log time series of stocks, which we obtained from Equation (7); $R_{\tau }^{x}=\left(r_{1}^{x},r_{2}^{x},\ldots ,r_{200}^{x}\right)$ and $R_{\tau }^{y}=\left(r_{1}^{y},r_{2}^{y},\ldots ,r_{200}^{y}\right)$ for all (X,Y) pairs. Further, we estimate the interaction information for all possible (X,Y,Z) triplets. Estimating these information-theoretic quantities is difficult as the number of sample points is finite, and the estimates are prone to sampling bias. Ref. [46] proposed a Gaussian copula-based estimator to overcome this sampling bias problem. We estimate mutual and interaction information using the gcmi Python package version 0.4 Python package [46]. Next, to check the statistical significance of the obtained values of the mutual and interaction information, we perform the *p*-test on the mutual information data with a *p*-value of 0.05. For the interaction information, we select only those having negative values because those are the three uniform hyperedges that are synergistic as per the definition of the interaction information.

We resort to using networks to study the structure of the dyadic interactions. Nodes of the network represent companies, and edges represent interactions between pairs of companies. The weight of an edge is given by the value of mutual information between the two companies’ log time series data. Further, as a general network extension, a hyperedge may have more than two nodes. If a hypergraph is constructed with each hyperedge containing a *d* number of nodes, it is referred to as a *d* uniform hypergraph. To capture the structure of synergistic or higher-order interactions, we proceed similarly as in [36]. We construct a 3-uniform hypergraph, where each hyperedge consists of 3 nodes whose interaction information is a negative value. To visualize the structure of pairwise interactions, we choose only the dominant pairwise interactions by choosing a threshold. The threshold is selected in the following manner: we plot a histogram of all the mutual information values that were found to be significant from the p-test, and an edge is added between two nodes only if the mutual information between those two nodes exceeds the average of all the mutual information values plotted in the histogram. Similarly, for the triadic interactions, we choose only dominant synergistic interactions. The threshold is chosen such that a hyperedge is added between three nodes only if the interaction information value of that particular triplet succeeds in the mean of the negative interaction information value distribution. We use the “extra-node” representation as in [47] to visualize the hypergraph when the number of hyperedges is large. This representation gives us a better visualization of the interactions than the usual Euler representation of the hyperedges.

## 4. Results and Discussion

While it can be the case that stocks belonging to the same sectors also have synergy, the redundant information dominates, and concluding that these stocks do not have synergistic interactions because interaction information is positive can be false, as interaction information measures the net synergy. We can conclusively infer that the three sets of stocks with a negative value of the interaction information are synergistic. Hence, essentially, we are capturing a subset of all the synergistic interactions. Nonetheless, our results indicate that new important information about the interaction structure is revealed when these synergistic interactions alone are considered. In the case of a *d* uniform hypergraph, the degree of a node is the number of hyperedges that the node is involved with.

We analyze the pairwise graphs and hypergraphs to comprehend what information hypergraphs yield that is not revealed by corresponding pairwise networks. We systematically analyze various network characteristics and compare mutual and interaction information with average volatility, the total number of edges, Forman–Ricci curvature, von Neumann entropy, and average eigenvector centrality for pairwise and higher-order hypergraphs. All these quantities are calculated for all the five markets for all the windows. The vertical lines in the figures denote crashes. Also, the time evolution of degree and hyperdegree and pairwise and higher-order eigenvector centrality for individual companies are analyzed. Also, to quantify the variability in quantity, we use the measured coefficient of variation for each quantity, such as the number of edges, Forman–Ricci curvature, and von Neumann entropy.

### 4.1. Average Mutual/Interaction Information and Average Volatility

For each stock market and each window, we construct the corresponding network by finding the mutual information values between each pair of nodes and hypergraph by finding the interaction information among each triplet of the nodes. At the time of a financial crash, stock prices in a market are expected to be correlated more [18]. As a result, the average value of mutual information increased during the crash periods of 2015, 2018, and 2020. At the same time, the interaction information, which is also dependent on mutual information, may or may not manifest any trend because we simultaneously need the synergistic part of the interaction information to be more than that of the redundant part of the interaction information, as explained earlier to detect statistically valid higher-order interactions.

**Figure 2:**
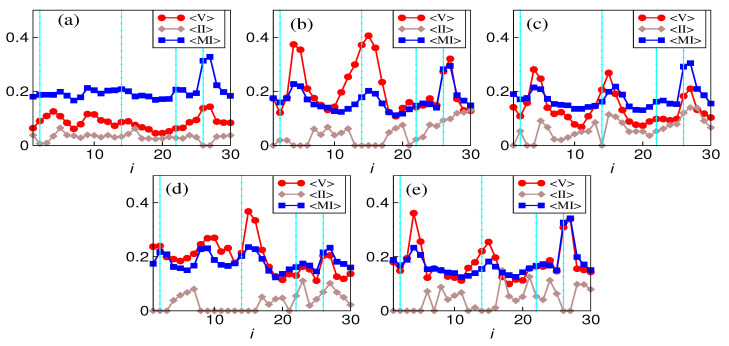
Comparison of magnitudes of average MI and average II with average volatility (standard deviation) of (**a**) BSE, (**b**) FTSE, (**c**) DAX, (**d**) NIKKEI, (**e**) SP500. The values of each quantity are scaled to make the comparison. Here, 〈MI〉 (blue), 〈II〉 (grey), and 〈V〉 (red) stand for the mean value of MI, II over all the edges, and volatility over all the stocks, respectively.

We analyze the average volatility (standard deviation), average mutual information, and average interaction information. Average volatility and average interaction information are scaled for the sake of the comparison. Figure 2 reflects that the average mutual information and average volatility behave similarly throughout all the 30 windows. In all the markets except the Indian one, the 15th window has a peak for volatility, which is closely followed by mutual information. They both attain local maxima, especially during the immediate window after the crash periods. Meanwhile, the average interaction information manifests no systematic, continuous trend with the window indices. Even then, in the vicinity of the 14th window, average interaction information has a very low value. One more observation from the five plots was that. In contrast, all the other markets showed significant variability in the average value of mutual information in the vicinity of the crashes, and the Indian market showed significant variability only during the COVID-19 crash. This is not a feature of the higher-order interactions but adds to the idea that developing markets behave differently.

These observations alone are insufficient to draw any conclusions regarding the behavior of both pairwise and higher-order networks during the period considered here. So, as the systematic continuation of the first step, we are looking at the number of pairwise and higher-order edges in the functional network during the period.

### 4.2. Time Evolution of Number of Edges

The stock prices vary with time, and consequently, the mutual information and interaction information values and the corresponding network structures do, too. Therefore, it is reasonable to assume that the variation in the number of edges might point to something that has happened in the market. It is also a good idea to look at both pairwise and higher-order networks separately to check whether higher-order networks can provide additional insights into the structure and evolution of the stock market as a complex system.

**Figure 3:**
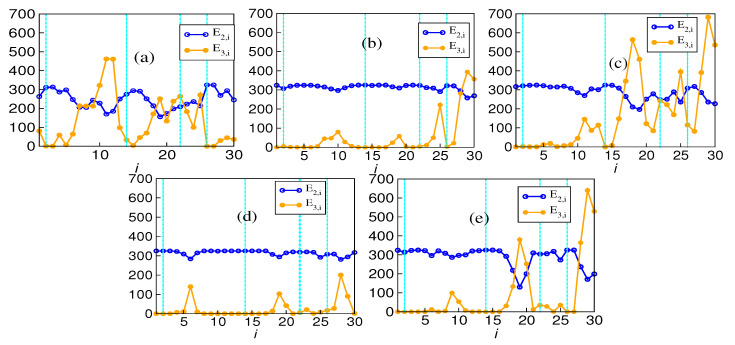
Number of pairwise $E_{2,i}$ (blue open circles) and hyperedges $E_{3,i}$ (orange solid circles) as a function of window index *i* for (**a**) BSE, (**b**) DAX, (**c**) NIKKEI, (**d**) NIKKEI, and (**e**) SP500. In each market except NIKKEI, there exists a peak around the 10th and 20th window, as well as also close to the 30th window.

We have plotted the number of edges against the window index for all five markets in Figure 3. The behavior of the number of edges (both pairwise and hypergraph) with the window index indeed reflects that the Indian market, being a developing market unlike the other four, behaves differently in terms of the total number of edges also. Even when the average mutual information has comparable variability to that of interaction information, the variability of pairwise edges is less than that of the hyperedges. This trend is more pronounced in the other four markets than in the Indian market. This shows the robustness of the pairwise structure in comparison to the higher-order structure. The two main observations from these graphs are that the hypergraph is sparse in the 14th window for all the markets. There has been a considerable rise in the number of hyperedges after the COVID-19 crash, with the Indian market still being the exception in both cases. Also, except for the Indian market, few peaks in the plots occur approximately during the same time, especially in DAX, NIKKEI, and SP500. The 14th window corresponds to the period of the 2015 crash. This leads to the inference that higher-order structures exist, at least in the three uniform hypergraph parts.

The number of higher-order edges attains a minimum at the 14th window for all the five markets. It is not likely to be a coincidence, given that during this time, a crash affected almost all the stock markets in the world. We have to look into why information about the interaction between stock prices is particularly low in this window for all the markets. The second observation is that the number of hyperedges increases sharply after the 26th window (COVID-19 crash). The crash of 2015 was endogenous, while 2020 was an exogenous one. As a result, the time series of the stock prices during these two periods are expected to differ.

The choice of the window length affects the mutual and interaction information, which are both statistical quantities obtained from the time series. To ensure that these differences do not alter the results, we have evaluated the variation in the number of edges through the period using three window sizes, namely 100 days, 200 days, and 400 days. Even though there are variations, the prominent peaks in the time evolution plot are sustained. Figure A4 shows the variation with the changing window size.

To quantify the extent of variability in the pairwise and higher-order structures, we calculated the co-efficient of variability for both and found that pairwise network is much more robust than that of its higher-order counterpart. For example, for BSE, the value of the pairwise coefficient of variability is 0.19, and for higher-order, it is 0.97, which clearly quantifies the robustness of the pairwise network.

### 4.3. Forman–Ricci Curvature

**Figure 4:**
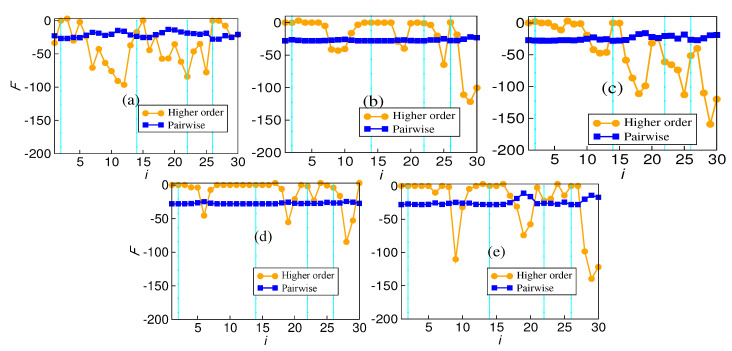
Forman–Ricci curvature *F* averaged over edges in all 5 markets for both networks and hypergraphs: (**a**) BSE, (**b**) DAX, (**c**) FTSE, (**d**) NIKKEI, (**e**) SP500.

The calculation of Forman–Ricci curvature for pairwise and higher-order networks also tells the same story as the number of edges. The variation in Forman–Ricci curvature is plotted against the window index in Figure 4. While significant variability exists in the higher-order Forman–Ricci curvature, pairwise interactions are more or less rigid. There is no correlation between the volatile periods and the value of either pairwise Forman–Ricci curvature or higher-order Forman–Ricci curvature. However, the variability in the Forman–Ricci curvature is higher in the case of higher-order than pairwise. For every market, the variability in the case of the hypergraph is much greater compared with that of the pairwise network. The results for the five markets are given in Table 1.

### 4.4. Von Neumann Entropy

**Figure 5:**
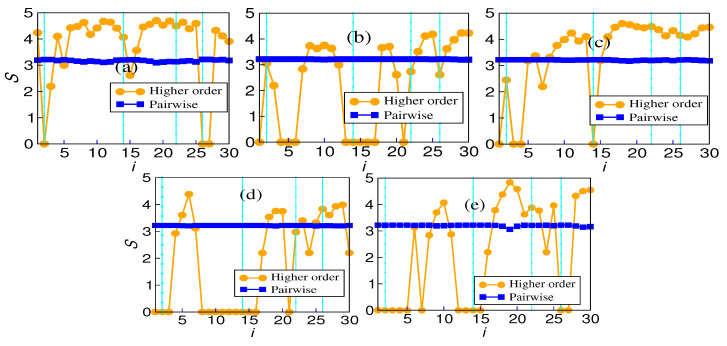
Von Neumann entropy *S* for the networks and hypergraphs corresponding to each window: (**a**) BSE, (**b**) DAX, (**c**) FTSE, (**d**) NIKKEI, (**e**) SP500.

Von Neumann entropy was also calculated for both pairwise and higher-order graphs for all the windows and all the markets as can be seen in Figure 5. The network structure drastically changes over time intervals, and as von Neumann entropy gives information about the network’s community structure [44], it also varies accordingly. Just like curvature, von Neumann’s entropy also shows more variability in the case of a higher order than that of pairwise networks. For example, in the case of BSE, CV is 0.01 for pairwise and 0.37 for higher order. The coefficient of variability behaves the same way for other markets also, as in Table 1.

## 5. Conclusions

**Figure 6:**
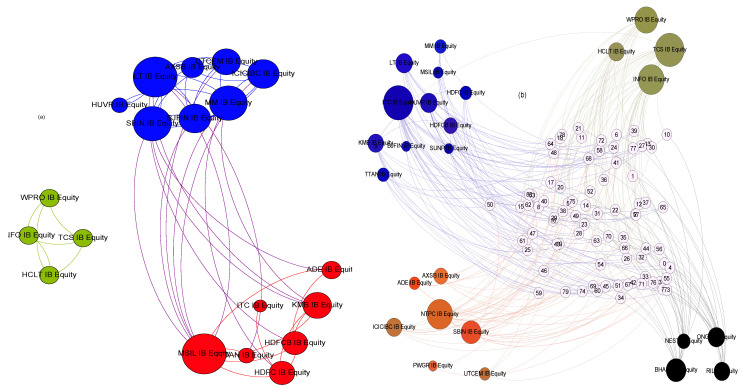
The visualization of the network structures: (**a**) pairwise, (**b**) higher order. The color is chosen so that for the pairwise network, members of the same community have the same color. The same color pattern is followed in the higher-order case, too. Extra node representation is used to represent higher-order interactions. Both pairwise and higher-order networks are for the 19th window (22 May 2017 to 26 February 2018) for BSE market.

We have proposed a method to construct networks and hypergraphs using information-theoretic quantities, namely mutual and interaction information, and provide a common framework for both two and three-unit interactions. It can be seen as a first step in extending the methodology to other higher-order interactions via O-information [48]. We start by analyzing the average values of mutual and interaction information to see their relationship with volatility. Average mutual information followed the same trend as average volatility; however, average interaction information does not reflect any systematic trend over time. Further, the mutual information and volatility have peaks near the crashes, whereas the Indian market has the same only during the COVID-19 crash. This disparity may be expected, as the Indian market is a developing market and is supposed to behave differently [5]. These observations and inferences could be drawn by analyzing the average mutual information, interaction information, and volatility (Figure 6).

**Figure 7:**
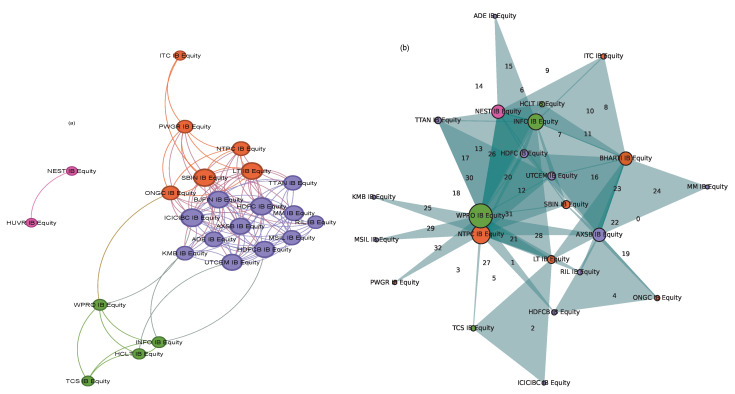
The visualization of the network structures: (**a**) pairwise, (**b**) higher order. The color is chosen so that for the pairwise network, members of the same community have the same color. The same color pattern is followed in the higher-order case, too. Both pairwise and higher-order networks are for the 27th window (1 June 2020 to 2 November 2020) for BSE market.

**Figure A1:**
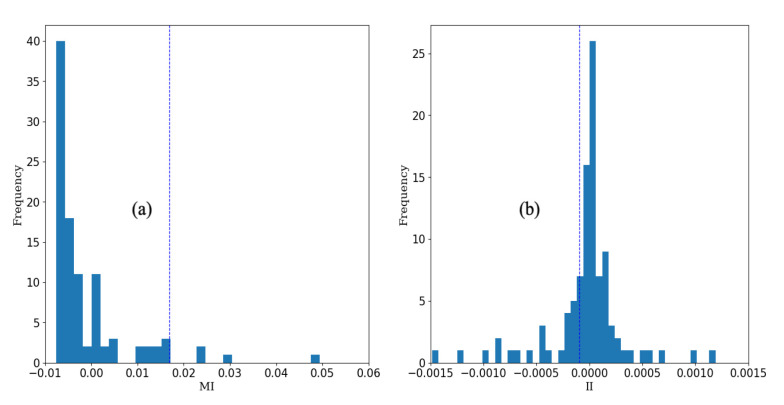
(**a**) Permutation test for mutual information. The blue line indicates 99 percent of distribution values. (**b**) The permutation test for interaction information.

**Figure A2:**
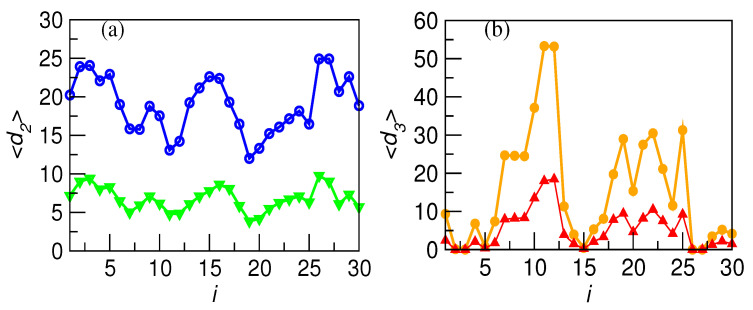
Impact of the threshold value: (**a**) $\langle d_{2}\rangle$ vs *i* (window index) before (open blue circles) and after the threshold (open green triangles); (**b**) $\langle d_{3}\rangle$ vsi *i* before (closed orange circles) and after (closed red triangles). All plots are for BSE stock.

**Figure A3:**
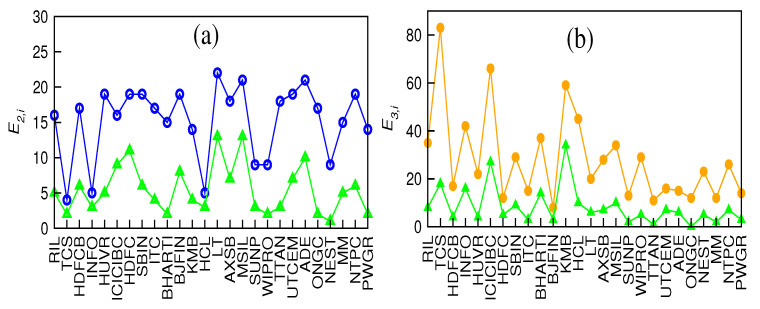
Effect of threshold on $d_{2,k}$ and $d_{3,k}$ for BSE stock for a particular window (say 20th window): (**a**) $d_{2,k}$ before threshold (open blue circle), after threshold (open green triangle), (**b**) $d_{3,k}$ before threshold (closed orange circle), and after threshold (closed green triangles).

**Figure A4:**
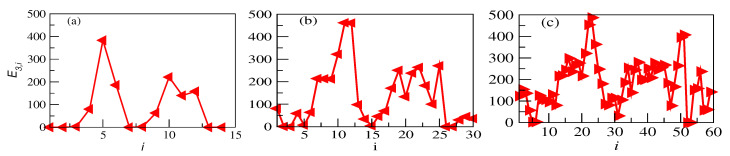
Dependence of window size on the network. The total number of 3-uniform hyperedges ($E_{3,i}$) is plotted against the window index (*i*) (**a**) for a window size of 400 and overlap 200, (**b**) for a window size of 400 and overlap 200, (**c**) for a window size 100 and overlap 50. This shows that even when a larger window size filters the fluctuations in the graph, it sustains the most pronounced peaks, showing that they are not numerical artifacts but the result of some phenomena that affected the stock markets during the time period. The graphs are plotted for the BSE stock market over a time period of 12 years.

The number of edges in the hypergraph may reflect something different because the average value may not tell the whole story. This analysis adds that the Indian market, being a developing market, unlike the other four, behaves differently regarding the total number of edges. The variability of pairwise edges is less in comparison to the hyperedges. This trend is more pronounced in the other four markets than in the Indian market. The absence of hyperedges around window 14 indicates the absence of higher-order structures surrounding a major endogenous crash. This is also reflected in the vicinity of other crashes. The surge in hyperedges after the COVID-19 crash is more pronounced. It is known that endogenous and exogenous crashes are fundamentally different, and their recovery rates differ. An exogenous crash’s recovery rate is faster than an endogenous crash’s. The variation in the number of edges reflects that higher-order interactions are more prevalent in this fast recovery period than in other crash periods. The plot of average interaction information also reflects the same behavior. It depicts a higher value after the COVID-19 crash than all the other crashes. Also, it is notable that the Indian market is a bit different in that it does not conform with other markets in the sudden surge. The Forman–Ricci curvature corresponding to the pairwise and higher-order networks is calculated from the individual degree of each node. The pairwise Forman–Ricci curvature is known to point to network fragility [20]. Looking into the higher-order version, as it depends on each node’s degree, higher-order Forman–Ricci curvature more or less follows the same behavior as the total degree. In DAX, FTSE, NIKKEI, and SP500, quite a few windows exist where the number of edges is zero. The von Neumann entropy in a pairwise network relates to the community structure, so it has a high value if the connections are uniformly distributed. When communities start forming, the corresponding value is less. However, in the case of higher-order interactions, no discernible pattern exists. No pattern exists during the crashes either (Figure 7).

The calculation of the coefficient of variability for different quantities like edge number and Forman–Ricci curvature shows that pairwise structure remains more unchanged in comparison with higher-order interactions. This time period witnessed multiple crashes and the consistently low value of the coefficient of variability for the pairwise networks points to its robustness against such events. A more in-depth study of the market to the level of individual firms and sectors incorporating higher-order interactions can provide further insights into this problem.

## Figures and Tables

**Table 1:** The coefficient of network variability for different quantities in the five markets. Here, *N* stands for number of edges, *F* for Forman–Ricci curvature, and *S* for von Neumann entropy. Each cell’s first and second entries are for pairwise networks and hypergraphs. In each column, *P* stands for pairwise and *H* stands for higher order.

Stock Market	*N*		*F*		*S*	
	P	H	P	H	P	H
BSE	0.19	0.97	0.19	0.77	0.01	0.37
DAX	0.05	1.96	0.05	1.59	0.002	0.752
FTSE	0.13	1.17	0.14	0.99	0.004	0.4
NIKKEI	0.042	2.06	0.03	2.02	0.008	0.92
SP500	0.18	1.93	0.19	1.65	0.01	0.82

## Data Availability

The original contributions presented in the study are included in the article, further inquiries can be directed to the corresponding author. The data used in this study are available through Bloomberg terminals.

## Abbreviations

The following abbreviations are used in this manuscript:

MDPI	Multidisciplinary Digital Publishing Institute
DOAJ	Directory of open-access journals
TLA	Three letter acronym
LD	Linear dichroism

## Appendix A

### Appendix A.1. Data

A detailed description of the data used is given in the data subsection. The specific details regarding the number of companies selected for the analysis from each market are provided in the first Table A1. Also, the information regarding the sliding windows and the corresponding date is provided in Table A2.

**Table A1:** Number of stocks. Here, *M* stands for the total number of companies in each market, and *N* stands for the number of companies we selected for our study. Companies were selected based on market capitalization, data availability, and diversity.

Stock Market	*M*	*N*
BSE	100	26
FTSE	100	26
DAX	50	26
NIKKEI	225	26
SP500	500	26

**Table A2:** Window index and the corresponding starting dates. *i* corresponds to the window index, and *T* corresponds to the starting date of each window. As there is an overlap, the window will be from the starting date to the starting date of the next to the next window. For example, the first window is from *i* = 1 to *i* = 3.

*i*	1	2	3	4	5	6
*T*	28/06/10	15/11/10	04/04/11	22/08/11	09/01/12	28/05/12
*i*	7	8	9	10	11	12
*T*	15/10/12	22/07/13	09/12/13	28/04/14	04/03/13	15/09/14
*i*	13	14	15	16	17	18
*T*	02/02/15	22/06/15	09/11/15	28/03/16	15/08/16	02/1/17
*i*	19	20	21	22	23	24
*T*	22/05/17	09/10/17	26/02/18	16/07/18	03/12/18	22/04/19
*i*	25	26	27	28	29	30
*T*	09/09/19	27/01/20	01/06/20	02/11/20	22/03/21	09/08/21

### Appendix A.2. Permutation Test

We randomly permuted the time series data and calculated the mutual and interaction information for each pair and triplet of companies, respectively. The process was repeated 100 times for each window. Only the mutual information values whose values from the randomized series were less than that of the original value for 95 percent of the iterations were saved. The other edges were discarded. In the case of hyperedges, the criteria were that the values should be greater than the original value for 95 percent of the iterations. The results obtained for the permutation test of the Mutual and Interaction information estimator for the time window are shown in Figure A1.

## Appendix B

### Appendix B.1. Effect of Thresholding and Window Size

The threshold was selected so that the strongest interactions were sustained while the others were discarded. However, to make sure that the application of the threshold does not affect the macroscopic parameters, we compared the networks with and without the threshold. Figure A2 shows the average pairwise and higher-order degree with and without threshold. Figure A3 plots the degree of each company in the network with and without a threshold.
